# Impact of Sedation Regimen on Diagnostic Adequacy in Endoscopic ultrasonography-Guided Tissue Acquisition of Solid Pancreatic Lesions

**DOI:** 10.1055/a-2888-0687

**Published:** 2026-06-19

**Authors:** Luciano Lenz, Silvia Kim, Bruno Martins, Leika Miyahara Felipe, Sergio E. Matuguma, Andressa Abnader Machado, Renata Nobre, Marcelo Simas de Lima, Gustavo A. de Paulo, Adriana V. Safatle-Ribeiro, Fauze Maluf-Filho

**Affiliations:** 1 Gastroenterology 215027 University of Sao Paulo Institute of Cancer of Sao Paulo StateSão PauloSão PauloBrazil; 2 Endoscopy 37896 Hospital Israelita Albert EinsteinSão PauloSão PauloBrazil; 3 Endoscopy 89577 Fleury GroupSão PauloSão PauloBrazil; 4 Anesthesiology 215027 University of Sao Paulo Institute of Cancer of Sao Paulo StateSão PauloSão PauloBrazil; 5 Anesthesiology 89577 Fleury GroupSão PauloSão PauloBrazil; 6 Pathology 215027 University of Sao Paulo Institute of Cancer of Sao Paulo StateSão PauloSão PauloBrazil; 7 Gastroenterology 164358 Conselho Nacional de Desenvolvimento Científico e TecnológicoBrasíliaDFBrazil

**Keywords:** endoscopic ultrasonography, pancreas, quality and logistical aspects, sedation and monitoring, fine-needle aspiration/biopsy

## Abstract

**Background and study aim** Endoscopic ultrasonography-guided tissue acquisition (EUS-TA) requires patient cooperation for optimal tissue yield. Sedation improves comfort and procedural conditions, but the impact of different sedation strategies on tissue adequacy is unclear. This study compared balanced propofol sedation (BPS) versus general anesthesia (GA) for EUS-TA of solid pancreatic lesions (SPLs) using propensity score matching (PSM).

**Patients and methods** Single-center, cohort study with retrospective analysis of a prospectively collected database. All patients undergoing EUS-TA for SPL from April 2013 to February 2025 were included. Standalone EUS was performed under BPS; combined EUS-ERCP procedures under GA. Anesthetist-assisted (AS) deep sedation (without intubation) was classified as. Adequacy was defined as sufficient material for cytologic/histologic interpretation (inadequate if only blood or epithelial cells). Direct comparisons, multivariable logistic regression, and PSM were performed.

**Results** In all, 491 EUS-TA procedures in 433 patients (50.3% female) were performed; the overall adequacy was 85.7%. BPS was used in 370 (74.5%, adequacy 84.6%), GA in 93 (18.9%, 89.2%), and AS in 28 (5.7%, 89.3%) ( *p* = 0.448). No significant difference between BPS and GA ( *p* = 0.256) or endoscopist-directed (BPS) vs. anesthetist-directed sedation (GA + AS) ( *p* = 0.209). Multivariable regression (adjusted for needle type, diagnosis, lesion location) showed no association of sedation method with adequacy (GA vs. BPS: *p* = 0.565). After PSM (needle type, adenocarcinoma, pancreatic head lesions), no significant differences emerged across modalities.

**Conclusions** In this large real-world cohort, BPS achieved comparable adequacy to GA for EUS-TA of SPL. Deeper sedation does not appear to improve adequacy in tertiary centers. Prospective studies on safety and cost-effectiveness are needed.

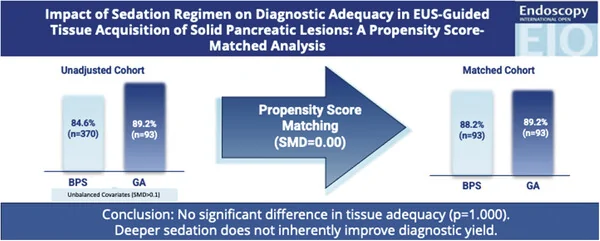

## Introduction

Pancreatic cancer is currently the 12th most common cancer and the 6th leading cause of cancer-related death globally, with an estimated more than half a million new cases and around 460 hundred deaths in 2022. The incidence of the disease continues to rise annually, with projections indicating almost twice as many new cases in 2050, potentially reaching a total of 1 million new cases in the world. 1

Pancreatic adenocarcinoma carries a poor prognosis, with a 5-year survival rate of less than 10%. The lethality of pancreatic cancer is largely due to the inherent difficulty of early detection, with only 10–20% of patients being diagnosed at a stage of resectable disease. 2 Early diagnosis may improve the outcome and survival in patients with pancreatic cancer. 3

Tissue acquisition is essential for diagnosis and therapeutic planning. Options include CT/US-guided biopsy, ERCP brush cytology, surgical biopsy, and Endoscopic Ultrasound-Guided Tissue Acquisition (EUS-TA), performed via EUS-FNA (fine-needle aspiration) or FNB (fine-needle biopsy). EUS-TA for solid lesions is well established, with EUS-FNA diagnostic yields ranging 71–100%. An inconclusive result on initial EUS-TA can potentially delay diagnosis and access to appropriate care. 3 4 5 6

As EUS is uncomfortable and time-consuming, sedation is necessary to improve patient satisfaction and procedure success rate. 7 The effect of the type of sedation on EUS-TA is poorly studied. 3 8 9 Non-anesthesiologist–administered propofol (NAAP), including nurse-administered propofol sedation (NAPS) and balanced propofol sedation (BPS), is safe and effective. NAPS uses propofol alone, whereas BPS combines an opioid/benzodiazepine dose followed by propofol. 10 11

Although sedation is routinely used for advanced endoscopic procedures (EUS and ERCP), practices vary internationally and include endoscopist-directed sedation and anesthesiologist-assisted anesthesia. Furthermore, the practice of anesthesiologist-assisted anesthesia is divergent and differs between monitored anesthesia care (MAC) performed without endotracheal intubation and general anesthesia (GA). 10 12

Ootaki et al. demonstrated that GA was associated with a significantly higher diagnostic yield of EUS-FNA when compared with conscious sedation (CS) with midazolam and meperidine or fentanyl. 3 However, whether GA is also better than BPS is unexplored.

Hence, the main aim of this study was to compare the adequacy of BPS and GA for EUS-TA for solid pancreatic lesions (SPLs). The secondary aims were to compare the adequacy when the sedation was performed by the endoscopist (BPS) versus by the anesthesiologist (GA + AS), and to compare the adequacy performed by deep sedation (BPS + AS) versus GA.

## Patients and Methods

This study was approved by our institutional review board (No. 88350625.7.0000.0068/NP 5294/95). This is a single-center, controlled cohort study, with retrospective analysis of a prospectively collected database including all patients submitted to EUS-TA for diagnosis of a solid pancreatic mass at the gastrointestinal endoscopy unit of Instituto do Cancer do Estado de São Paulo (ICESP)—a tertiary referral cancer center in Brazil from April 2013 to February 2025. An advanced endoscopic fellow usually performed the endosonographic study under the supervision of an expert endoscopist.

Patients who presented with SPLs based on previous imaging studies were included. Exclusion criteria included patient age <18 years and pancreatic cystic lesions.

### EUS–TA

All TAs were performed under EUS guidance using a linear echo-endoscope. The best track for puncture was chosen after careful examination using the Doppler mode. Next, EUS-TA was performed under real-time echographic visualization.

The Fanning technique was used in all the procedures. At least two needle passes were performed per procedure. The first was usually performed using the slow-pull technique, and the subsequent ones using dry suction. During the slow-pull technique, the stylet was progressively and constantly withdrawn throughout the to-and-fro motions. For the dry suction technique, a negative pressure of 5–20 mL was used. The needle was moved back and forth around 20 times per pass.

From 2020 to 2022, 50 patients were enrolled in a clinical trial (NCT NCT04877340) comparing standard 22-gauge FNA with the 22-gauge Franseen in which the same lesion was punctured by both needles. 13 Before this protocol, all EUS-TAs were performed by FNA needle and after that all of them were done with a 22-gauge FNB.

Rapid-on-site evaluation (ROSE) was not available. Specimens were placed in formaldehyde 10% bottle for cell block evaluation. According to the discretion of the endoscopist, the material was sent on slides in absolute alcohol as well. A certified pathologist, who was unaware of the sedation/anesthesia method, analyzed the material obtained from the punctures.

### Sedation/Anesthesia Regimens

Electrocardiogram, pulse oximetry, and noninvasive blood pressure were monitored in both groups.

1. **General Anesthesia (GA)**
  According to our institutional protocol, all ERCP is performed under GA. Many patients with pancreatic cancer present with jaundice, thus requiring endoscopic biliary drainage, and it is common for ERCP and EUS to be performed concomitantly. Patients who underwent ERCP and EUS on the same day under GA, as well as some cases in which the EUS was performed alone but under orotracheal intubation, were analyzed in the group GA. GA is also based on the need for airway protection and mechanical ventilation, deep neuromuscular blockade, and uncomfortable patient positioning.
  Board-certified anesthesiologists with extensive experience in endoscopic sedation were responsible for the GA. GA was administered using cisatracurium (0.15 mg/kg) as a muscle relaxant and propofol (1.5–2 mg/kg) for induction. The depth of anesthesia was maintained using an inhalational anesthetic, sevoflurane, with a 50% mixture of oxygen and air. In addition, the anesthetist used fentanyl (2–3 mcg/kg) and other multimodal anesthesia agents at their discretion.
  The procedures (ERCP and EUS) were performed with the patients in the prone position.
2. **Balanced Propofol Sedation (BPS)**
  In this group, a single dose of fentanyl (50 mcg) followed by a dose of midazolam (5 mg) was administered, followed by titrated doses of propofol at the discretion of an experienced endoscopist (when patients exhibited symptoms of discomfort).
  The patients were positioned lying on their left side before sedation. A nursing technician administered the medications, provided procedural assistance, and remained beside to assist the patient as needed (i.e., oropharyngeal suctioning). All patients received nasal oxygen supplementation (2–4 L/min).
3. **Anesthesiologist Sedation (AS)**
  If the procedure was not accompanied by ERCP and the patient had severe comorbidities, a certified anesthesiologist was also responsible for sedation (AS). However, unlike the GA group, neither orotracheal intubation nor neuromuscular blockers were used. AS would entitle deeper levels of sedation, or use other classes of anesthesia agents, such as ketamine, lidocaine, or alpha-2 agonists (dexmedetomidine).

### Outcome Measures

The sample was considered adequate if sufficient material was obtained for cytologic/histologic interpretation, and inadequate if sufficient cells were absent, i.e., aspirates with only blood or gastrointestinal trajected epithelial cells. We defined adequacy as the proportion of samples for which a final pathologic diagnosis could be made. 14 A sample was defined as malignant if it was either positive or suspected of malignancy. A negative or atypical sample was considered benign. 15 Therefore, malignancy was defined as the sum of final diagnoses of adenocarcinoma, neuroendocrine tumors, metastases, lymphomas, and Frantz tumors. Benign lesions were those without malignancy, with a pathologic diagnosis of inflammatory process. Safety was evaluated based on the occurrence of major procedural adverse events. Given the retrospective nature of the data, minor and transient adverse events (e.g., self-limiting hypotension or bradycardia) were not consistently documented and were thus excluded from the analysis.

### Data Collection

All endoscopic procedures in our service are recorded into an electronic data file. From this database, all EUS were filtered and then all TA-EUS of solid lesions of the pancreas were manually selected, excluding cystic lesions and extra-pancreatic punctures. These selected cases were transferred to Red Cap.

Relevant variables were extracted from medical chart, endoscopy, pathology, and anesthesiology records.

### Statistical Analysis

Categorical data were expressed as proportions, and continuous data were expressed as mean with standard deviation (SD). The Fisher or chi-squared test for categorical variables and *t* test or nonparametric Wilcoxon signed-rank test for continuous variables were used where appropriate to compare baseline demographics and sedation/anesthesia modalities. The statistical significance was considered for *p* < 0.05. Social Science Statistics website was used for statistical analyses ( https://www.socscistatistics.com/ ).

Statistical analysis was performed to compare adequacy rates among the study groups (BPS, GA, and AS). Categorical variables were analyzed using Pearson’s chi-square test, while Fisher’s Exact test was applied when expected cell frequencies were less than 5. Odds ratios (ORs) and relative risks (RRs) were calculated with 95% confidence intervals (95% CI). A *p* -value <0.05 was considered statistically significant. To adjust confounding factors, multivariable logistic regression models were constructed. The calibration and fit of the models were rigorously assessed using the Hosmer and Lemeshow goodness-of-fit test, where a *p* -value >0.05 indicated an adequate model fit. ORs with 95% confidence intervals (CIs) were calculated to identify independent predictors of sample adequacy, with a significance level set at *p* < 0.05. Statistical analysis was performed to compare adequacy rates among the procedure groups (BPS, GA, and AS). For the comparison of categorical variables, Pearson’s chi-square test was used. Fisher’s Exact test was applied when expected cell frequencies were less than 5 to ensure accuracy. To identify independent predictors of sample adequacy, multivariate logistic regression models were constructed. These models included variables with a *p* -value <0.20 in the univariate analysis or recognized clinical relevance, such as sedation group, needle type, and histologic nature. Two distinct models were used: Model A adjusted for adenocarcinoma, and Model B adjusted for general malignancy (including adenocarcinoma, neuroendocrine tumors, metastases, lymphoma, and Frantz tumors).

Propensity score matching (PSM) was performed using *R* statistical software (version 4.5.2) with the MatchIt package to ensure a balanced comparison between sedation groups.

## Results

A total of 491 EUS-TAs were performed in 433 patients included in this study. The mean age was 62 years (range 25–87), with a slight predominance of women (50.3%). The mean lesion size was 36 mm (range 2–120 mm), and most lesions were in the pancreatic head (72.7%). Reflecting the oncologic profile of the center, adenocarcinoma accounted for 71.9% of final diagnoses. Most procedures were performed with a fine needle (FNA) (412, 83.9%). Overall sample adequacy was 85.7% (95% CI 82.3–88.6%). In all 370 procedures (75.4%) were performed under BPS, 93 (18.9%) under GA, and 28 (5.7%) under AS (1.8%). Baseline demographic and lesion characteristics are summarized in Table 1 .

**Table 1:** Patients and lesion characteristics.

	Group			
Characteristic	Endoscopist	Anesthetist		
	BPS	GA	AS	Overall
Patients ( *n* )	328	93	25	433
Procedures ( *n* )	370	93	28	491
Age (mean - range)	61 (25–86)	62 (27–84)	64 (56–69)	62 (25–86)
Sex (female %)	50.6%	48.4%	60.0%	50.3%
				
Lesion location *n* (%)				
Head	250, 67.6%	87, 93.5%	20, 71.4%	357, 72.7%
Uncinate	62, 16.8%	21, 22.6%	3, 10.7%	86, 17.5%
Neck	66, 17.8%	8, 8.6%	3, 10.7%	77, 15.7%
Body	84, 22.7%	8, 8.6%	7, 25.0%	99, 20.2%
Tail	40, 10.8%	5, 5.4%	2, 7.1%	47, 9.6%
				
Size of lesion (mm)	35 (2–90)	37 (2–120)	36.3 (11.5–100)	36 (2–120)
				
Final diagnosis				
Adenocarcinoma	255, 68.9%	79, 84.9%	19, 67.9%	353, 71.9%
Neuroendocrine	43, 11.6%	5, 5.4%	0, 0.0%	48, 9.8%
Metastasis	14, 3.8%	2, 2.2%	5, 17.9%	21, 4.3%
Inflammatory	46, 12.4%	3, 3.2%	3, 10.7%	52, 10.6%
Neoplasia intraductal mucinosa	5, 1.4%	1, 1.1%	0, 0.0%	6, 1.2%
Tumor de Frantz	3, 0.8%	1, 1.1%	0, 0.0%	4, 0.8%
Lymphoma	3, 0.8%	1, 1.1%	1, 3.6%	5, 1.0%
Others	1, 0.3%	1, 1.1%	0, 0.0%	2, 0.4%
				
Needle				
FNA	316, 85.4%	80, 86.0%	16, 57.1%	412, 83.9%
FNB	16, 4.3%	6, 6.5%	10, 35.7%	28, 5.7%
Both	38, 10.3%	7, 7.5%	2, 7.1%	47, 9.6%

BPS—balanced propofol sedation.

GA—general anesthesia.

AS—anesthetist sedation.

FNA—fine-needle aspiration.

FNB—fine-needle biopsy.

All procedures, regardless of the group, were completed successfully. Regarding procedural safety, no major adverse events directly related to the EUS-TA, such as perforation or hemorrhage requiring blood transfusion, were observed in any of the sedation groups.

Sample adequacy was 84.3% (95% CI 80.6–88.0%) in the BPS group, 89.2% (95% CI 83.0–95.2%) in the GA group, and 89.3% (95% CI 78.8–100%) in the AS group ( *p* = 0.448). Direct comparison between BPS and GA did not reach statistical significance ( *p* = 0.256). Similarly, no difference was observed when comparing endoscopist-directed sedation (BPS) with anesthetist-directed sedation (GA + AS) ( *p* = 0.209). Additionally, adequacy rates did not differ significantly between procedures performed under tracheal intubation (GA) versus those conducted under with nonintubated sedation (BPS and AS groups combined) ( *p* = 0.296) ( Table 2 ).

**Table 2:** Results adequacy according sedation group.

Comparison	Adequacy (%)	*p* -Value	OR (95% CI)	RR (95% CI)	Test
**BPS vs. GA vs. AS**	84.6% vs 89.2% vs 89.3%	0.448	N/A	N/A	Chi-square
**BPS vs. GA + AS**	84.6% vs 89.3%	0.209	1.51 (0.80–2.86)	1.06 (0.98–1.13)	Chi-square
**BPS + AS vs. GA**	84.9% vs 89.2%	0.296	1.47 (0.72–2.98)	1.05 (0.96–1.15)	Chi-square
**BPS vs. GA**	84.6% vs 89.2%	0.256	1.51 (0.74–3.08)	1.05 (0.97–1.15)	Chi-square
**BPS vs. AS**	84.6% vs 89.3%	0.759	1.51 (0.45–5.04)	1.05 (0.91–1.22)	Fisher’s Exact
**AS vs. GA**	89.3% vs 89.2%	1.000	1.00 (0.25–4.00)	1.00 (0.86–1.16)	Fisher’s Exact

BPS, balanced propofol sedation; GA, general anesthesia; AS, anesthetist sedation; OR, odds ratio; RR, relative risk.

To identify independent predictors of sample adequacy and to control for potential confounders, multivariable logistic regression analysis was performed. Two distinct models were evaluated to assess the impact of different histologic classifications while adjusting for sedation groups and needle types. In both models of multivariate logistic regression analysis, sedation method was not associated with sample adequacy. Although GA showed a trend toward higher adequacy compared to BPS in both Model A (adenocarcinoma as outcome: adjusted odds ratio [aOR] 1.50; 95% CI 0.71–3.08; *p* = 0.295) and Model B (malignancy as outcome: aOR 1.56; 95% CI 0.75–3.22; *p* = 0.230), these associations did not reach statistical significance. Similarly, AS did not yield a significant advantage over BPS. Independent predictors of sample adequacy were lesion located in the pancreatic head (aOR 2.14, *p* = 0.022) and malignant histology (aOR 2.21, *p* = 0.019) ( Table 3 ). Both models showed an excellent fit according to the Hosmer–Lemeshow goodness-of-fit test.

**Table 3:** Multivariable logistic regression models for sample adequacy.

Variable	Model A (Adenocarcinoma)	Model B (Malignancy ^1^ )
	aOR (95% CI)/ *p* -value	aOR (95% CI)/ *p* -value
Sedation group		
Endoscopist (BPS)	Reference	Reference
Anesthetist (AS)	1.41 (0.40–4.95)/0.589	1.44 (0.41–5.02)/0.565
General anesthesia (GA)	1.50 (0.71–3.08)/0.295	1.56 (0.75–3.22)/0.230
Needle type		
FNA	Reference	Reference
FNB	1.19 (0.57–2.48)/0.641	1.17 (0.58–2.40)/0.658
Location		
Others (body/tail/neck)	Reference	Reference
Head of pancreas	2.08 (1.08–3.99)/0.029	2.14 (1.11–4.13)/0.022
Histological nature		
Benign/inflammatory	Reference	Reference
Target group	1.92 (1.05–3.51)/0.033	2.21 (1.14–4.28)/0.019

^1^ Malignancy includes adenocarcinoma, neuroendocrine tumor, lymphoma, metastasis, and Frantz’s tumor.

aOR: adjusted odds ratio. Fit assessed by Hosmer–Lemeshow test (Model A *p* = 0.634; Model B *p* = 0.834).

PSM was performed using a nearest-neighbor 1:1 approach without replacement, stratified by sedation type (sedation without intubation [BPS/AS] vs. GA). Covariates included needle use, lesion location affecting the pancreatic head, and presence of adenocarcinoma. After matching, an excellent balance between groups was achieved, with standardized mean differences below 0.1 for all covariates ( Figs. 1–3 ) ( Table 4 ). Once balance was confirmed for key variables (needle type, adenocarcinoma diagnosis, and lesions in the pancreatic head), no statistically significant differences in sample adequacy were observed across the sedation modalities, including those with (GA) versus without intubation (BPS + AS), anesthesiologist-led (GA + AS) versus endoscopist-led sedation (BPS), and GA versus BPS— Table 5 .

**Fig. 1:**
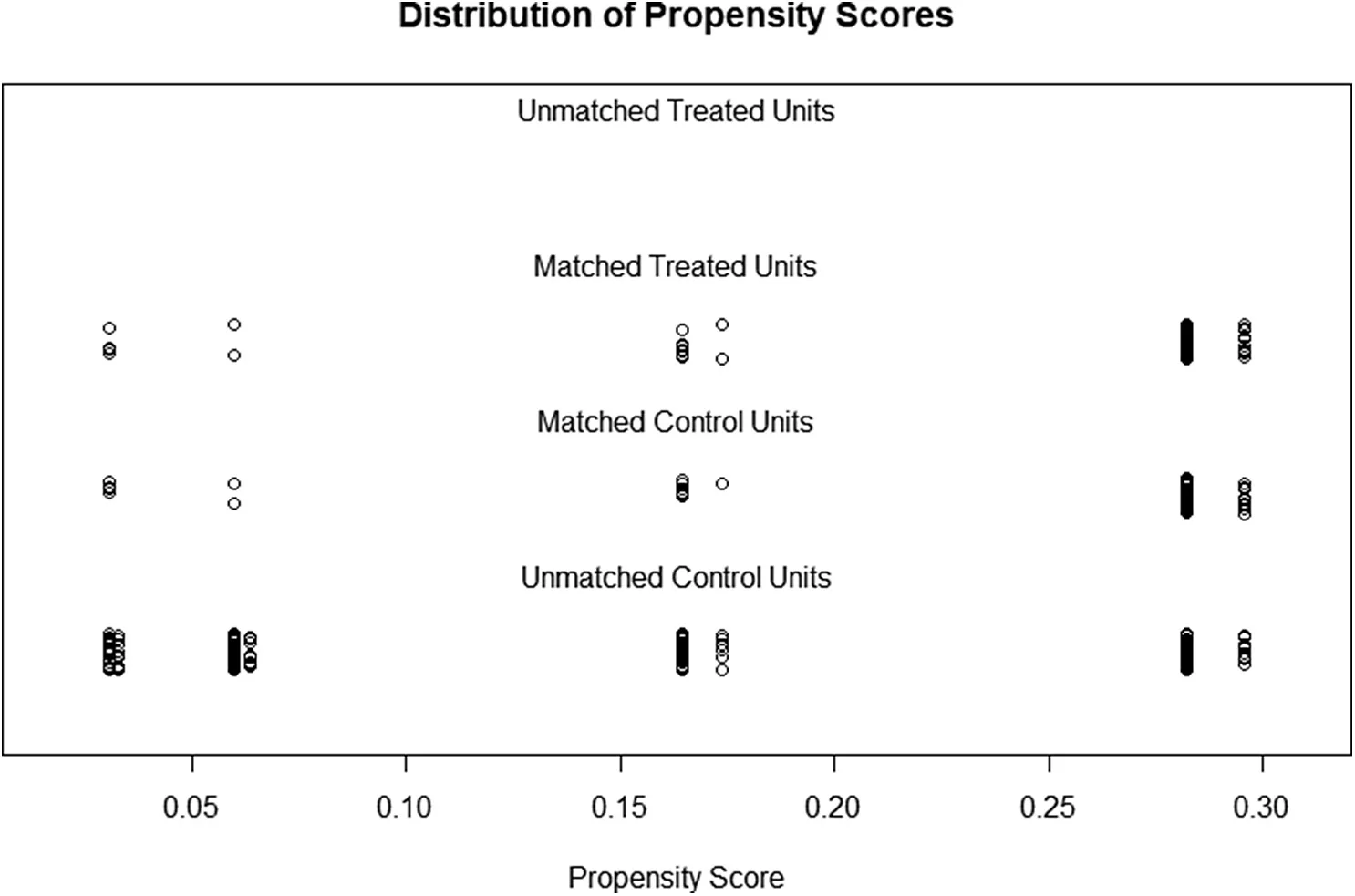
Jitter plot of propensity scores BPS vs. GA.

**Fig. 2:**
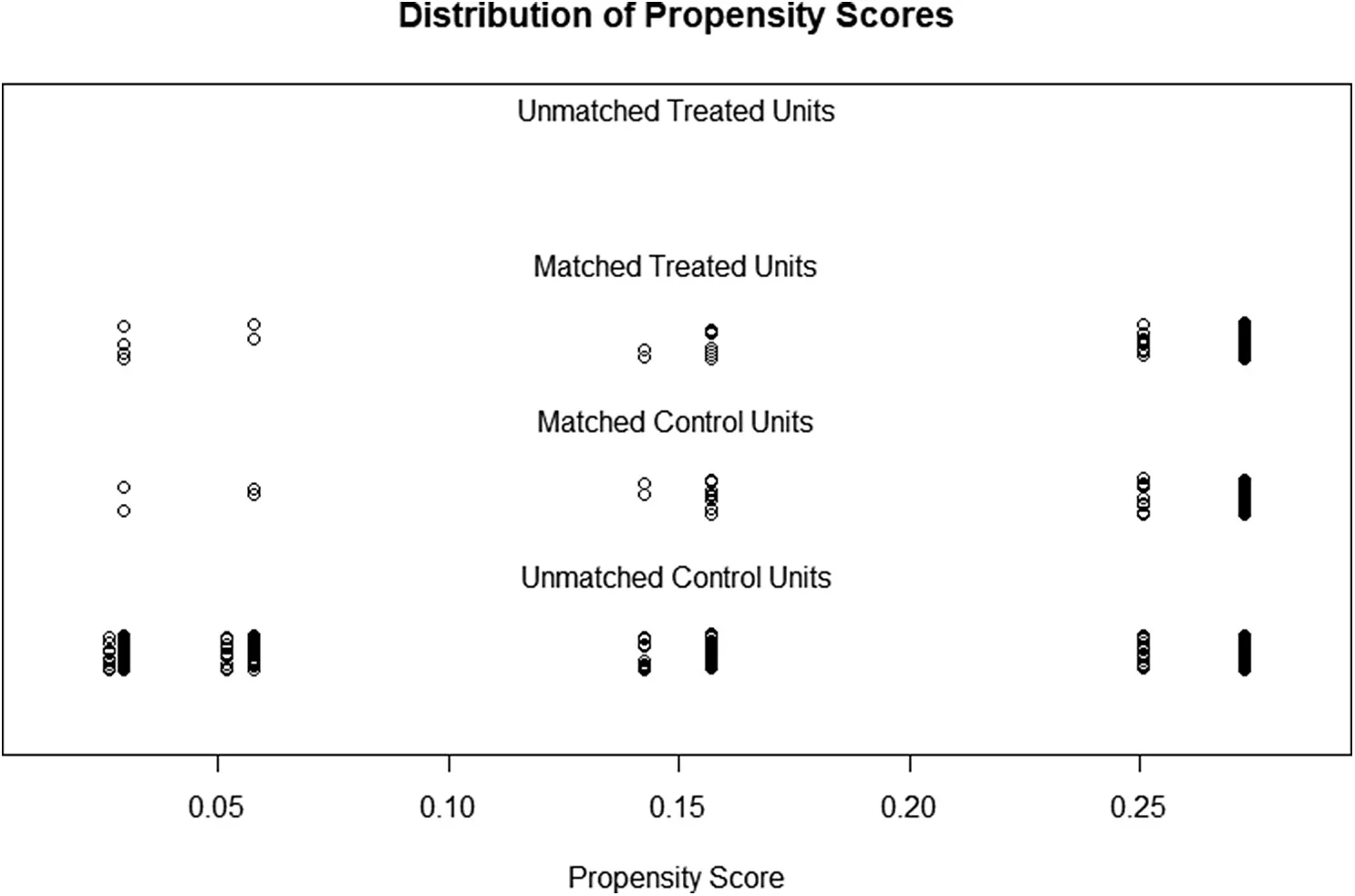
Jitter plot of propensity scores BPS + As vs. GA.

**Fig. 3:**
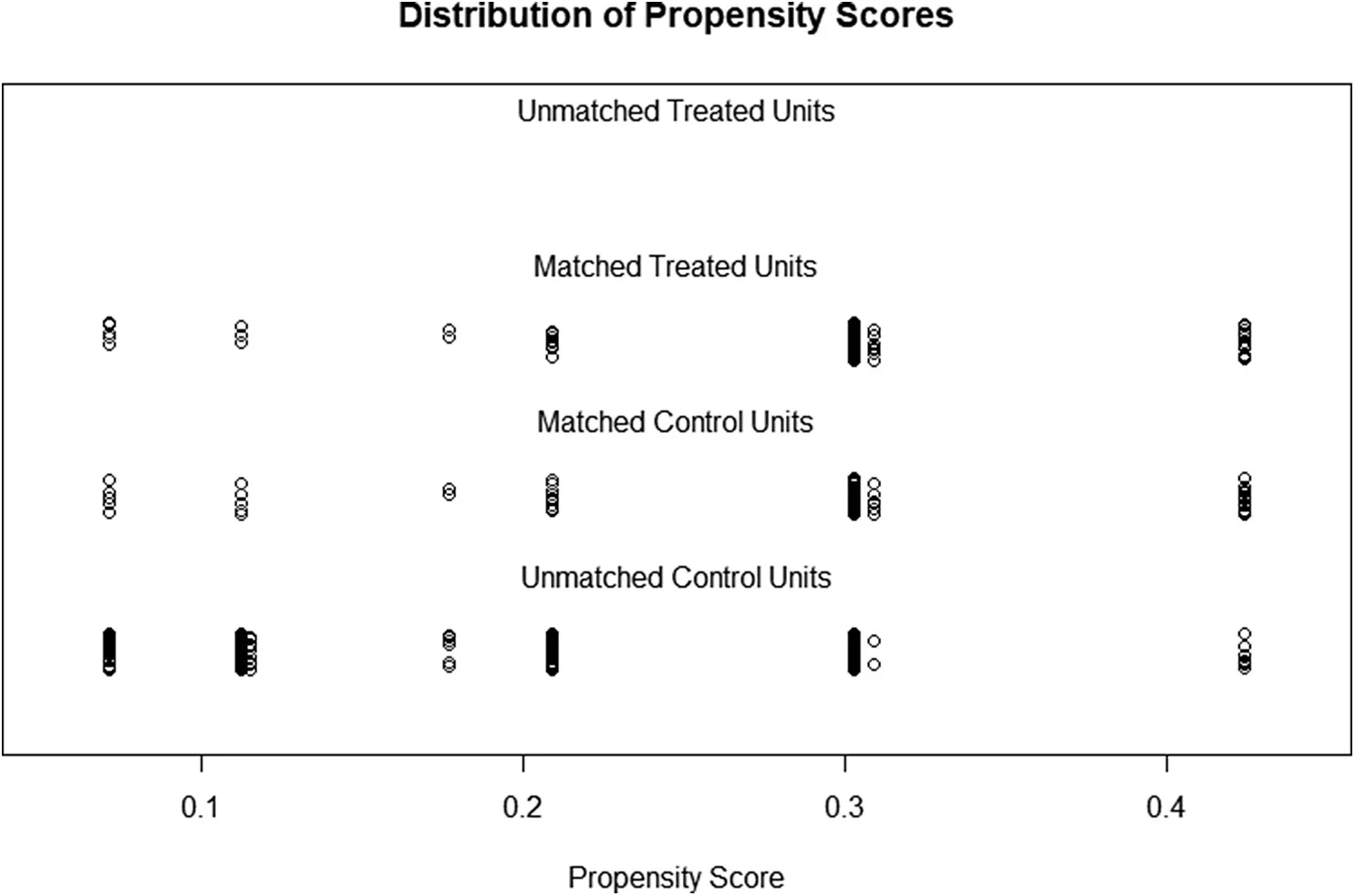
Jitter plot of propensity score BPs vs. GA + AS.

**Table 4:** Baseline characteristics before and after propensity score matching.

Covariates/groups	Before matching (unadjusted)		After matching (adjusted)	
	BPS ( *n* = 370)	GA ( *n* = 93)	BPS ( *n* = 93)	GA ( *n* = 93)
**Needle type (FNA)**	316 (85.4%)	80 (86.0%)	80 (86.0%)	80 (86.0%)
**Adenocarcinoma**	255 (68.9%)	79 (84.9%)	79 (84.9%)	79 (84.9%)
**Head location**	250 (67.6%)	87 (93.5%)	87 (93.5%)	87 (93.5%)
**SMD** *	–	**0.36**	–	**0.00**

*SMD: standardized mean difference.

Values <0.10 indicate adequate balance between groups.

**Table 5:** Sample adequacy before and after propensity score matching.

Status	BPS group	GA group	*p* -Value
**Before matching (unadjusted)**	**84.6%**	**89.2%**	**0.256**
( *n* /total)	(313/370)	(83/93)	

**After matching (adjusted)**	**88.2%**	**89.2%**	1.000*
( *n* /total)	(82/93)	(83/93)	

Status	BPS group	GA + AS group	*p* -Value
**Before matching (unadjusted)**	**84.6%**	89.3%*	0.205*
( *n* /total)	(313/370)	(108/121)	

**After matching (adjusted)**	**87.6%**	**89.2%**	**0.840**
( *n* /total)	(106/121)	(108/121)	

Status	Non-intubated (BPS + AS)	Intubated (GA)	*p* -Value
**Before matching (unadjusted)**	**84.9%**	**89.2%**	**0.286**
( *n* /total)	(338/398)	(83/93)	

**After matching (adjusted)**	**88.2%**	**89.2%**	1.000*
( *n* /total)	(82/93)	(83/93)	

BPS—balanced propofol sedation.

GA—general anesthesia.

AS—anesthetist-sedation.

## Discussion

In this study, the different sedation/anesthesia regimens had similar results in the adequacy of EUS-TA for SPLs. Although a minor numerical difference was observed, GA does not confer any meaningful advantage in terms of adequacy even in the academic scenario when the procedure was performed by a fellow in training. The theoretical benefit of the patient being completely immobile during GA was not reflected in the findings. Probably, an endoscopist trained in sedation is sufficient for the puncture to obtain an adequate sample, even if the patient moves occasionally. Consequently, BPS delivered by endoscopists could be considered the standard of care. Shaha et al. also found no statistical difference ( *p* = 0.0738) in technical success (adequate obtaining of ultrasound-guided puncture material in a wide range of indications) between endoscopist-guided sedation (CS) and care provided by an anesthesia care provider (ACP), including monitored anesthesia care (MAC) or general anesthesia (GA) administered by an anesthesiologist or certified registered nurse anesthetist. 16 On the other hand, in the work of Ootaki et al., GA (83%) was associated with a successful diagnosis as compared with CS (73%) ( *p* = 0.01). 3 However, both Shaha and Ootaki used sedation by an endoscopist consisting of a combination of an opioid and a benzodiazepine (CS) without propofol, whereas in our study, the endoscopists also used propofol. 3 16 Propofol has a favorable pharmacokinetic profile with rapid elimination due to this short half-life. Patient satisfaction with propofol sedation for endoscopic procedures is equivalent or superior to that of CS. Propofol administration during EUS and ERCP is more cost-effective than CS. 12 17 18 19 20 21

Propofol is a powerful, fast-acting sedative traditionally restricted to anesthesia professionals. Its use by non-anesthesiologists—particularly gastroenterologists during endoscopy—has generated international debate involving safety, legal, economic, and professional concerns. However, large international datasets and multiple guideline statements indicate that NAAP can be safe and effective when performed by properly trained personnel under structured protocols, with adverse event rates comparable to anesthesiologist-administered propofol. Gastroenterology societies in the United States jointly endorse NAAP based on evidence of safety, efficiency, and cost-effectiveness, whereas European guidelines support NAAP only in carefully selected patients and recommend anesthesiologist involvement for those with higher ASA class or elevated airway risk. 22 23 24 25 26 Finally, in the specific context of EUS, propensity-matched data show similar adverse event rates between NAAP and anesthesiologist-administered propofol even during complex procedures. 27

While propofol monotherapy is a widely used and effective standard for gastrointestinal endoscopy due to its rapid onset and offset, our center utilizes BPS to leverage the synergistic effects of combined agents. This approach allows for a significant reduction in the total propofol dose required, potentially enhancing hemodynamic stability. Indeed, research has shown fewer hypotensive episodes with BPS compared to propofol monotherapy (8% vs. 28%, *p* = 0.009). 28 Furthermore, the addition of an opioid provides necessary analgesia for high-stimulus procedures, which propofol alone lacks, and may result in shorter recovery and discharge times due to the lower cumulative dose of hypnotics. 29 Our clinical experience aligns with the findings of Pagano et al. who concluded that BPS administered by non-anesthesiologists is a safe and successful protocol for patients undergoing EUS-FNA. 11

Our findings suggest that while more profound sedation levels, such as GA, are often perceived to facilitate complex endoscopic procedures by reducing patient movement and improving procedural stability, they did not translate into a significant increase in sample adequacy for pancreatic EUS-FNA/FNB. The adjusted odds ratios for GA and AS were consistently above 1.0, suggesting a potential clinical trend. However, the lack of statistical significance indicates that the technical success of obtaining an adequate sample may be more dependent on lesional characteristics and anatomic location than on the type of sedation/anesthesia. These results imply that endoscopist-directed sedation remains a viable and effective option for maintaining adequacy, provided that procedural safety and patient comfort are maintained. Future studies with larger cohorts may be necessary to determine if the slight trend observed in favor of anesthetist-sedation becomes significant in more challenging technical scenarios.

It is estimated that anesthesia assistance in endoscopic procedures adds around 5 billion dollars per year to the health care costs in the United States. 30 The economic burden of EUS-TA can be partially explained by sedation-related costs. 31 Anesthetist-led sedation carries a prohibitive cost for universal application in EUS-guided procedures, reinforcing the importance of our findings that endoscopist-directed sedation provides comparable adequacy.

In our practice, GA was reserved for EUS-TA concomitant with ERCP. Performing tandem procedures starting with EUS followed by ERCP is logical and is being done at many centers. 32 Combined EUS and ERCP can be performed under the same sedation with similarly high yields for diagnostic EUS-TA. Benefits of a combined procedure may include expedited patient evaluation and the avoidance of repeated sedation. 33 According to the study by Sun et al., 34 which evaluated several associated factors, no association was found between the concomitant performance of EUS-TA and ERCP with isolated ultrasound-guided puncture. These authors also found no association between the presence of obstructive jaundice and bile duct dilatation (which are obviously more common in the ERCP group). However, they found a relationship between the type of needle, tumor size, and the location of the lesions with the outcomes of EUS-TA. In our study, the gender and the size of the lesions were quite similar between the groups. However, in the GA group, there were more cases of lesions affecting the head of the pancreas than in the BPS group. For this reason, a PSM was performed comparing the groups. Even after PSM for needle type, pancreatic head lesions, and final adenocarcinoma diagnosis, no statistically significant differences were found across the sedation modalities.

This study has some strengths. To our knowledge, this is the largest series directly comparing sedation and anesthesia regimens for obtaining cellular material through EUS-TA. In addition, previous studies compared CS with GA. BPS may be a more current regimen than CS. We also made other assessments, comparing who was responsible for sedation (endoscopist vs anesthetist) and with and without intubation. A significant strength of this study is its high external validity, derived from a diverse patient cohort in a tertiary cancer center. Furthermore, the methodologic rigor was enhanced using multivariate logistic regression and PSM, providing a “one-to-one” head-to-head, which ensured a balanced comparison and minimized selection bias by adjusting for key clinical confounders such as needle type, adenocarcinoma diagnosis, and lesion location. Another strength of this study is the histologic diversity of the included cohort, which extends beyond common ductal adenocarcinoma to encompass more rare and diagnostically challenging entities, such as neuroendocrine tumors, lymphomas, Frantz’s tumors, metastases, and pancreatic plasmacytoma. 13 35 36 37 Another strong point is that even though this was a retrospective study, there were no missing data.

This study had numerous important limitations. This was a retrospective single-center study conducted at a tertiary academic center, which limits the generalizability and leaves the possibility of unaccounted confounding variables. GA is driven by concomitant ERCP, which itself correlates with pancreatic head location, obstructive jaundice, and advanced disease. Although addressed via PSM and subanalysis, this remains a central source of confounding. Additionally, while this study did not measure specific procedural durations or recovery times, these factors are critical for evaluating clinical efficiency. Consequently, we have noted that both procedure and recovery times should be addressed in future prospective clinical trials to better define the impact of sedation depth on the overall workflow. Our study has certain limitations regarding safety data. While major adverse events were absent across all cohorts, the retrospective design prevented a detailed assessment of minor, transient adverse events. It is worth noting that while orotracheal intubation is a planned component of the GA protocol, its absence as an emergency requirement in the nonintubated groups (BPS and AS) further supports the feasibility of endoscopist-directed sedation in this setting. It is important to acknowledge that our results were obtained in a setting where ROSE is not routinely available. Procedures involving ROSE are typically longer in duration due to the immediate cytologic assessments required, which may interact differently with sedation depth compared to our cohort. Consequently, our findings regarding the comparability of sedation regimens may not be fully generalizable to clinical services where ROSE is standard practice. This study also did not evaluate the satisfaction of the endoscopist and patient. Finally, cost-effectiveness analysis was beyond the scope of the current study.

Looking toward future trends in sedation for advanced endoscopy, newer pharmacologic agents may further optimize procedural conditions. Specifically, remimazolam, an ultra-short-acting benzodiazepine, has emerged as a promising alternative that may offer a more stable hemodynamic profile compared to traditional agents. Recent studies have suggested the potential superiority of remimazolam over propofol and midazolam regarding safety and efficacy during EUS examinations. 38 39 Furthermore, the results of an ongoing prospective clinical trial specifically comparing remimazolam and propofol for EUS-TA are eagerly awaited to further clarify the role of this agent in enhancing diagnostic yield and patient safety. 40

## Conclusions

Despite such limitations, our analysis provides robust evidence supporting the favorable adequacy regardless of the sedation/anesthesia regimen used. Similar results were obtained between GA and BPS, as well as sedation performed by an anesthetist and by the endoscopist, with and without orotracheal intubation. Further randomized-controlled trials (RCTs) are warranted to confirm these findings.
